# The Akt/mTOR pathway: Data comparing young and aged mice with leucine supplementation at the onset of skeletal muscle regeneration

**DOI:** 10.1016/j.dib.2016.08.013

**Published:** 2016-08-11

**Authors:** Richard A. Perry, Lemuel A. Brown, David E. Lee, Jacob L. Brown, Jamie I. Baum, Nicholas P. Greene, Tyrone A. Washington

**Affiliations:** a Department of Health, Human Performance, and Recreation, University of Arkansas, USA; b Department of Food Science, University of Arkansas, Fayetteville, AR, USA

**Keywords:** MTOR, Skeletal muscle, Regeneration, Leucine supplementation, Aging

## Abstract

The data described herein is related to the article “Differential Effects of Leucine Supplementation in Young and Aged Mice at the Onset of Skeletal Muscle Regeneration” [1]. Aging is associated with a decreased ability of skeletal muscle to regenerate following injury. Leucine supplementation has been extensively shown, in young subjects, to promote protein synthesis during regeneration; however, the effects of leucine supplementation on the Akt/mTOR pathway in aged mice at the onset of muscle regeneration are not fully elucidated. In this article, we present data on the Akt/mTOR protein synthesis pathway at the onset of muscle regeneration in young and aged C57BL/6J mice that are and are not receiving leucine supplementation. More specifically, protein content of total Akt, mTOR, p70S6K and 4EBP-1 are presented. Additionally, we provide relative (phosphorylated:total) protein content comparisons of these targets as they present themselves in young and aged mice who have neither been injured nor received leucine supplementation. Lastly, markers of atrophy (FoxO1/O3, MuRF-1, Atrogin-1) are also reported in these young and aged control groups.

**Specifications Table**

**Table:** 

Subject area	*Biology*
More specific subject area	*Skeletal muscle, leucine supplementation, regeneration, aging*
Type of data	*Graphs, images*
How data was acquired	*Immunoblotting, PCR*
Data format	*Analyzed*
Experimental factors	*Three-month and 24-month old female C57BL/6J mice received an intramuscular injection of bupivacaine or PBS and were or were not given leucine supplementation.*
Experimental features	*Young and aged female C57BL/6J mice received an intramuscular injection of PBS or bupivacaine in the tibialis anterior. After the injection, mice were given or not given leucine supplementation. Three days after the injection, mice were sacrificed, and the tibialis anterior was isolated, weighed and prepared for immunoblotting.*
Data source location	*University of Arkansas, Fayetteville, Arkansas*
Data accessibility	*All data are provided with this article*

**Value of the data**

• The data provides age-induced alterations in protein degradation and synthesis markers which gives further insight into the natural physiological changes that occur as a function of age.
• Provides data on the effects of leucine supplementation on Akt/mTOR signaling proteins at the onset of skeletal muscle regeneration in an aged population which is valuable for future studies examining later time points when regeneration should be resolved.
• This data will help other researchers determine the effectiveness of leucine supplementation for promoting regeneration in an aged population.

## 1 Data

**Fig. 1:**
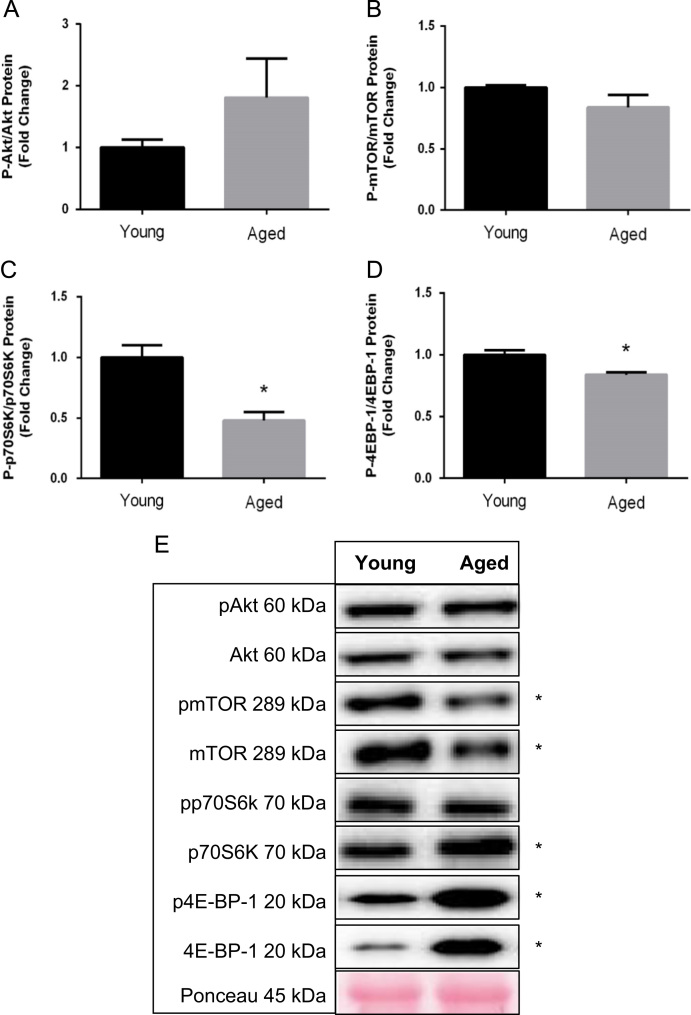
The effect of age on markers of protein synthesis in uninjured, untreated mice. A) p-Akt:Akt B) p-mTOR:mTOR C) p-p70S6K:p70S6K D) p-4EBP-1:4EBP-1 E) Representative blots of phosphorylated and total protein for each target. Significant difference is indicated by “*”. *P*≤0.05.

**Fig. 2:**
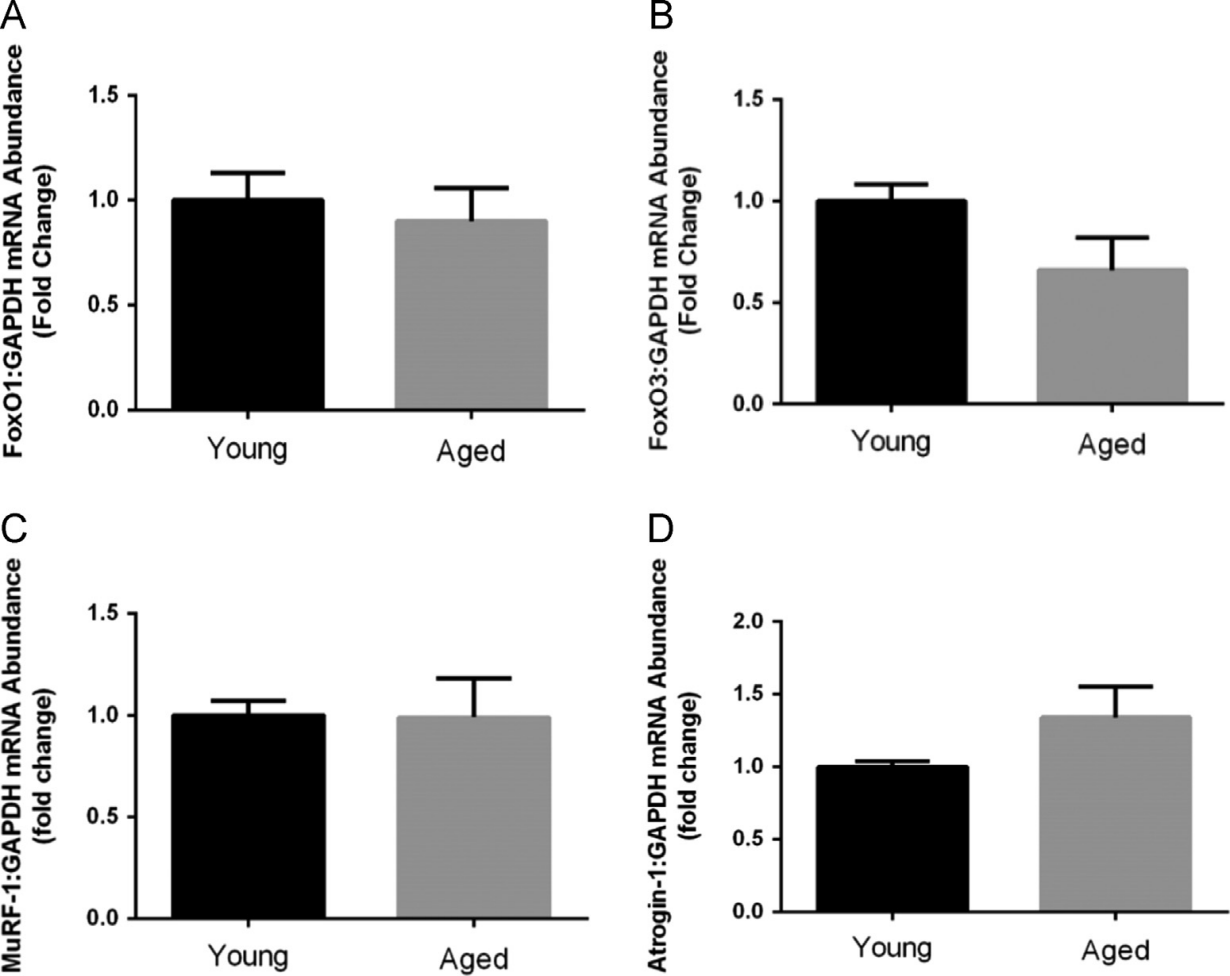
The effect of age on markers of protein degradation in uninjured, untreated mice. A) FoxO1:GAPDH B) FoxO3:GAPDH C) MuRF-1:GAPDH D) Atrogin-1:GAPDH. Significant difference is indicated by “*”. *P*≤0.05.

**Fig. 3:**
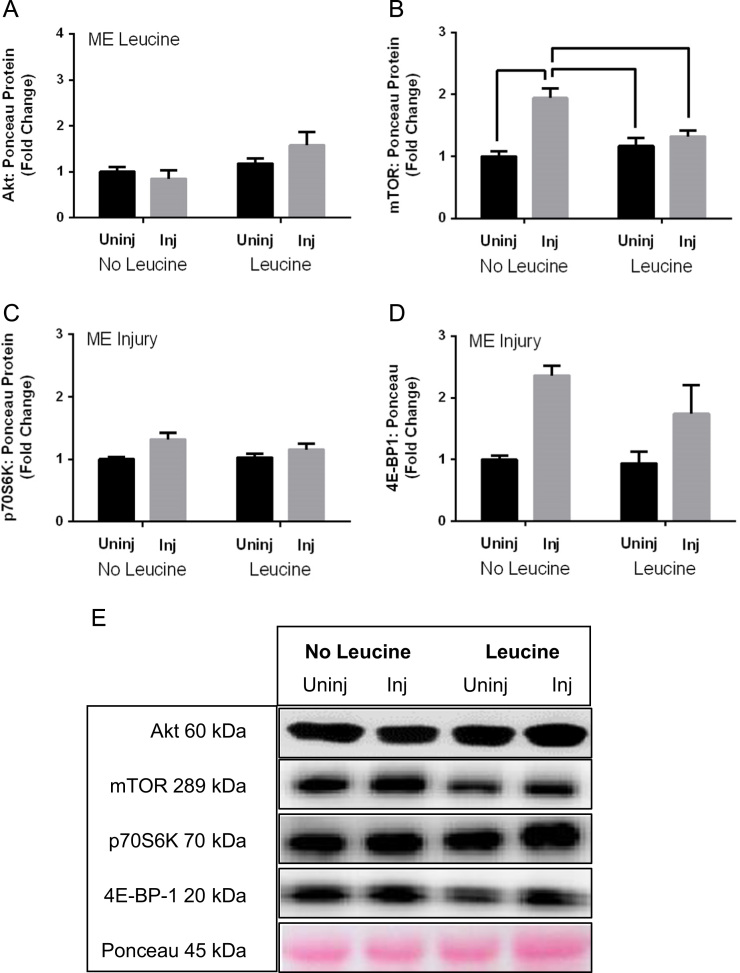
Total content of targets in the Akt/mTOR protein synthesis pathway in young mice at the onset of skeletal muscle regeneration. A) Akt B) mTOR C) p70S6K D) 4E-BP-1 E) Representative Blot. Main effect of injury is indicated by “ME Injury”, main effect of leucine is indicated by “ME Leucine”, and differences between groups are indicated by bars. *P*≤0.05.

**Fig. 4:**
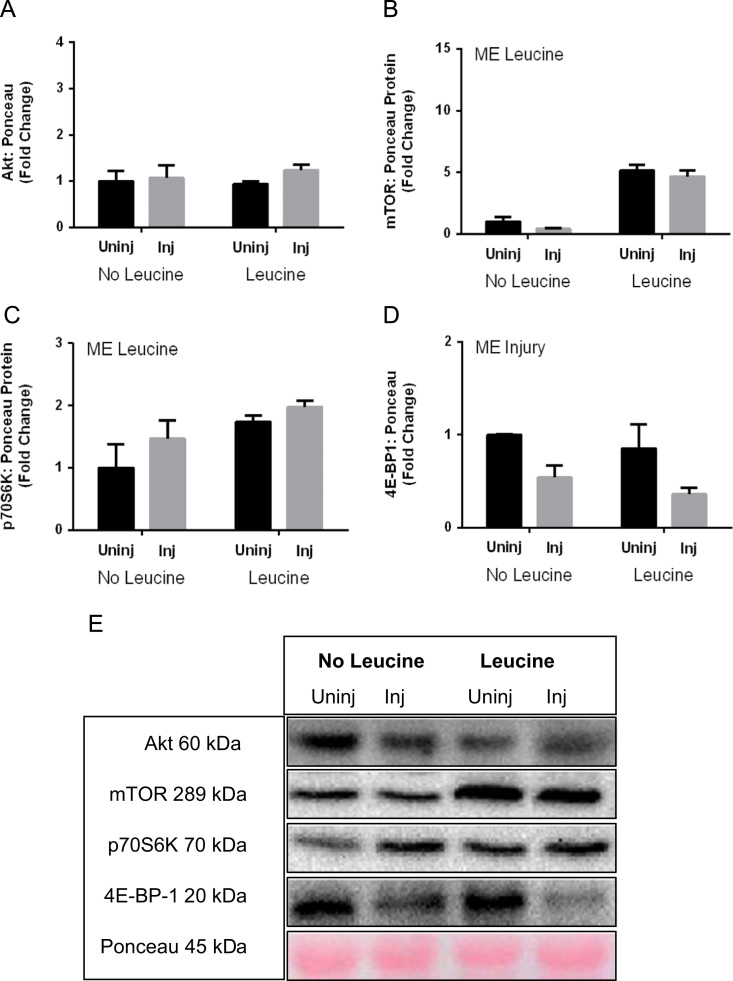
Total content of targets in the Akt/mTOR protein synthesis pathway in aged mice at the onset of skeletal muscle regeneration. A) Akt B) mTOR C) p70S6K D) 4E-BP-1 E) Representative Blot. Main effect of injury is indicated by “ME Injury”, and a main effect of leucine is indicated by “ME Leucine”. *P*≤0.05.

Fig. 1 contains western blot data of p-Akt/Akt, p-mTOR/mTOR, p-p70s6k/p70s6K, and p-4EBP-1/4EBP-1 in young and aged mice. Fig. 2 contains real time PCR data of FoxO1, FoxO3, MuRF-1, and Atrogin-1 in young and aged mice. Fig. 3, Fig. 4 contain western blot data of Akt, mTOR, p70s6K, and 4EBP-1 at the onset of skeletal muscle regeneration with or without leucine supplementation in young and aged mice, respectively.

## 2 Experimental design, materials and methods

### 2.1 Experimental design

Female C57BL/6J mice were raised to 3 months or 24 months of age and were injected with either 0.03 mL of phosphate buffered solution (PBS) or 0.75% bupivacaine (Marcaine) in the belly of the tibialis anterior (TA). After being injected, PBS- and bupivacaine-injected mice received leucine-infused water (1.5 g/100 mL) or normal drinking water [2], [3]. In total, both young and aged mice were divided into the following four groups: 1) no leucine/uninjured (*n* = 6); 2) no leucine/injured (*n* = 6); 3) leucine uninjured (*n* = 6); 4) leucine/uninjured (*n* = 6).

### 2.2 Muscle extraction and sample preparation

Three days post-injection, the TA and tibias were extracted as previously described [1], [4], [6]. The TA was snap frozen in liquid nitrogen and stored at −80 °C. Immediately following tissue harvest, the mouse was euthanized via exsanguination of the heart.

### 2.3 Immunoblotting analyses

Western blot analysis was performed as previously described [1], [4], [5]. Equal amounts of protein were loaded into 8-12% SDS-PAGE gels, subjected to electrophoresis, and transferred to a PVDF membrane. Blocked membranes were incubated with the following primary antibodies: p-Akt (Ser473), Akt, p-mTOR (Ser2448), mTOR, p-p70S6K (Thr389), p70S6K, p-4E-BP1 (Thr37/46), and 4E-BP1 (Cell Signaling, Danvers, MA). Anti-rabbit (7074) and anti-mouse (7076) secondary antibodies (Cell Signaling, Danvers, MA) were diluted 1:1000 to 1:2000 in 5% BSA or non-fat milk, in TBST, and incubated at room temperature for 1 h. Protein bands were detected with Enhanced Chemiluminescence (ECL) and the Fluorochem M Imager (Protein Simple, Santa Clara, California). The Ponceau-stained membranes were digitally scanned. The 45-kDa actin bands were quantified by densitometry and used as a protein loading correction factor for each lane as previously described [4], [5], [6].

### 2.4 Polymerase chain reaction analyses

RNA was extracted with Trizol reagent (Life Technologies, Grand Island, NY, USA) and reverse transcribed to cDNA as previously described [1], [4], [5], [7]. Real-time PCR was performed and results were analyzed using the StepOne Real-Time PCR detection system (Applied Biosystems, Foster City, CA). Commercially available Taqman probes (Applied Biosystems, Foster City, CA) were used for the following gene targets: FoxO1 (FAM), FoxO3 (FAM), MuRF-1 (FAM), Atrogin-1 (FAM), GAPDH (FAM). Cycle threshold (Ct) was determined and final quantification of gene expression was calculated using the ΔΔCt method {Ct = [ΔCt(calibrator) – ΔCt(sample)]}. Relative quantification was then calculated as 2^−ΔΔCt^.

### 2.5 Statistical analyses

Results are reported as mean ±SE. Pre-planned comparison between 3 month and 24 month uninjured, untreated controls (no injury, no leucine) were conducted by Student׳s *t*-tests. Two-way ANOVAs (leucine supplementation × injury) were conducted at each level of age to determine significant main effects and interactions (CA, SPSS 23). Post hoc analysis on significant interactions was done with a Student–Newman–Keuls test. Significance was established at an alpha level of 0.05.

## Funding

This work was supported by the Claude Pepper Older Americans Independence Center (P30 AG028718).
